# Medicine and Politics

**Published:** 1919-01-11

**Authors:** 


### The Hospital

The Workers' Newspaper of Administrative Medicine and Institutional Life, Administration, National Insurance and Health.

No. 1701, Vol. LXV. SATURDAY, JANUARY 11, 1919. PRICE TWOPENCE

### MEDICINE AND POLITICS.

The ambition to establish in Parliament a medical party has not received much encouragement from the recent elections. It is true that there is some slight increase in the number of those members of the House of Commons who hold a medical qualification, but this is a result rather of chance than of organised effort. A few of the medical men elected hold a considerable professional distinction and this has doubtless contributed to their success, more particularly in the University contests; and some of the re-elected members have established a more or less secure reputation as politicians. The remainder possess, it must be supposed, local personal or party claims and have come in with the flowing tide. In no instance can it be confidently claimed that the successful candidate sits as a member for medicine, and it may be doubted whether any of them received any appreciable help from a professional organisation. Certainly none of them revealed any precise views on the various questions which constitute medical politics, and neither the profession generally nor any considerable section of it can feel certain that a voice adequate to define and uphold medical interests has been secured. In short, for all practical purposes medical representation in the House of Commons remains *in statu quo*. There has been some change of *personnel*, but now, as formerly, the medical members have a political rather than a professional status in the House and none of them carries the endorsement of any particular professional organisation or party.

Such a result of the election is a striking contrast to the agitation and activities of recent months. The professional journals and certain corporate agencies have for months urged the need for medical representation and this more particularly in view of the probability of legislation likely to affect the interests of the profession. The argument has been placed in part on the claim for self-protection, but mainly on the ground of the public welfare. If, it is said, the health of the people is recognised as a concern of national importance and if, further, this aim is to be promoted by legislation, it is essential that Parliament shall include among its members men who can speak on this topic with expert authority, as only in this way can reliable guidance be secured. The most enthusiastic advocates of this doctrine went so far as to demand the creation of one or more medical constituencies in which doctors would exercise a special franchise and would secure a special and direct representation. Less ambitious was an organisation which hoped, by helping to finance medical candidatures, to secure representatives who would respond to professional claims, who would feel bound to assert these claims as opportunity offers. Last of all was an *ad hoc* committee called into existence at a meeting of the profession held in London and charged with the duty of furthering the cause of medical representation in the country generally. As a minor effort may be mentioned a curious body born far south of the Tweed, yet calling itself the Scottish Universities Parliamentary Association; its good will was directed to the success of a single medical candidate whose name appeared later at the bottom of the poll.

When the results of the election are examined it becomes evident that these various medical movements and enterprises practically came to no effective end. Something must be allowed, no doubt, for the peculiar circumstances of the election. The haste of the business afforded little time for effective organisation, and, more important still, the commanding and dramatic nature of the main issues necessarily overshadowed any class or professional ambitions. If has often been said that the electorate can only take an interest in a single question, and certainly it is not surprising that in the circumstances which surrounded the recent election the question of the presence of doctors in the House of Commons should fail to make any considerable appeal. A consideration of this order must doubtless have weighed with the various committees which undertook the task of promoting professional ambitions, and from what has been said these, like certain other causes unfortunate in the contest, evidently think that their time will come with the by-elections. It is a solace which has been cherished by many defeated candidates and no one grudges them whatever measure of comfort may be extracted from it. Here we have never professed to believe that the best interests of the medical profession will be secured by the organisation of a medical Parliamentary party, and we therefore contemplate the present position without any particular regret. There are various circumstances which make it difficult for representative practitioners to gain election to the House of Commons and the suggestion that special facilities ought to be provided to secure such representation we regard as a vain dream. On the other hand, the power of the profession and its influence on legislation were its members only agreed and organised would be enormous, and, to put the claim at its lowest, without this organisation and agreement medical members of the House of Commons would have no influence beyond that which is attached to their political and personal values. The lesson of the recent election in so far as it arises from the failure of the profession to send special representatives to Parliament is not therefore that efforts in this direction should be continued and improved. It is rather that the profession should strive to obtain a corporate mind. The means of effectively expressing this mind will inevitably follow, and whether in or out of Parliament the voice will be one of commanding importance. But so long as disunion and disagreement distinguish the professional ranks it is idle to expect attention and much less to sigh for authority and influence.

